# The relation between Ashworth scores and neuromechanical measurements of spasticity following stroke

**DOI:** 10.1186/1743-0003-5-18

**Published:** 2008-07-15

**Authors:** Laila Alibiglou, William Z Rymer, Richard L Harvey, Mehdi M Mirbagheri

**Affiliations:** 1 Sensory Motor Performance Program, Rehabilitation Institute of Chicago, Chicago, USA; 2 Interdepartmental Neuroscience Program, Northwestern University, Chicago, USA; 3 Department of Physical Medicine and Rehabilitation, Northwestern University, Chicago, USA

## Abstract

**Background:**

Spasticity is a common impairment that follows stroke, and it results typically in functional loss. For this reason, accurate quantification of spasticity has both diagnostic and therapeutic significance. The most widely used clinical assessment of spasticity is the modified Ashworth scale (MAS), an ordinal scale, but its validity, reliability and sensitivity have often been challenged. The present study addresses this deficit by examining whether quantitative measures of neural and muscular components of spasticity are valid, and whether they are strongly correlated with the MAS.

**Methods:**

We applied abrupt small amplitude joint stretches and Pseudorandom Binary Sequence (PRBS) perturbations to both paretic and non-paretic elbow and ankle joints of stroke survivors. Using advanced system identification techniques, we quantified the dynamic stiffness of these joints, and separated its muscular (intrinsic) and reflex components. The correlations between these quantitative measures and the MAS were investigated.

**Results:**

We showed that our system identification technique is valid in characterizing the intrinsic and reflex stiffness and predicting the overall net torque. Conversely, our results reveal that there is no significant correlation between muscular and reflex torque/stiffness and the MAS magnitude. We also demonstrate that the slope and intercept of reflex and intrinsic stiffnesses plotted against the joint angle are not correlated with the MAS.

**Conclusion:**

Lack of significant correlation between our quantitative measures of stroke effects on spastic joints and the clinical assessment of muscle tone, as reflected in the MAS suggests that the MAS does not provide reliable information about the origins of the torque change associated with spasticity, or about its contributing components.

## Introduction

Spasticity, a complex phenomenon, is one of the major sources of disability in neurological impairment including stroke. Spasticity is routinely defined as a motor disorder characterized by velocity-dependent increase in tonic stretch reflexes (muscle tone) with exaggerated tendon jerks, resulting from hyper excitability of the stretch reflex as one component of the upper motor neuron syndrome [1].

However, spasticity may involve complex changes in both neural and muscular systems, beyond a velocity dependent reflex resistance alone. Various alterations in musculo-tendinous structure such as alterations in muscle fiber size and fiber type distributions and probably fiber length, together with changes in mechanical and morphological properties of intra- and extra-cellular materials may also contribute to spasticity [2-5]. In the current study, we explore whether our objective measurements of neuromechanical abnormalities in the presence of spasticity are well-correlated with clinical assessments of spasticity (Modified Ashworth).

Despite spasticity being an important clinical problem, there is no universally accepted clinical measure of spasticity. Rating scales like the Ashworth scale (AS) and the Modified Ashworth scale (MAS) are the most commonly used clinical measures of spasticity but have clear limitations. For example, earlier studies have shown that these scales (Ashworth and Modified Ashworth) have a measurable but weak association with results from reflex-related EMG parameters (*Ashworth scale:*[6-11]*; Modified Ashworth scale: *[12-15]). But their association with objective measures of resistance to passive movement is stronger (*Ashworth scale:*[6,16-23]*; Modified Ashworth scale:*[23-31]). Therefore, the Ashworth scale may be regarded as a potentially useful clinical assessment of resistance to passive motion.

One potential problem of both the AS and the MAS is that these scales do not indicate if the resistance is due to a hyperactive stretch reflex, or whether it results from increased visco-elasticity of other tissues surrounding the joint. The Ashworth scales are unable to separate the contribution of different components of the neuromuscular system, or to determine which factors contribute under different functional conditions (such as different joint angles and different joint movement velocities). This differentiation is important, since it helps us to characterize the nature and origins of mechanical abnormalities associated with spasticity – these remain fundamental issues in our field. This information is also valuable for diagnosis and therapy, as these components arise from different physiological mechanisms.

Recently, we have developed a novel system identification technique [32-35] that enables us to characterize joint dynamic stiffness, and to separate the relative contributions of muscle, passive tissues and reflex action to overall joint stiffness. This study sought to determine whether there was a systematic relation between clinical measures of spasticity, notably the MAS, and quantitative measures of neuromuscular response to broad band position perturbations delivered to the ankle and elbow joints of the hemiparetic subjects (in both paretic and non-paretic limbs).

## Methods

This investigation was part of a cohort study designed to investigate the nature and origins of neural and mechanical abnormalities following a hemispheric stroke.

### Subjects

For our ankle study, twenty individuals with a single hemispheric stroke (59.2 ± 9.9 years) and for the elbow study, fourteen individuals with stroke (56 ± 12.7 years) with the similar inclusion criteria were recruited from the clinical outpatient department at the Rehabilitation Institute of Chicago (RIC). All the subjects gave informed consent to the experimental procedures, which had been reviewed and approved by the Institutional Review Board of Northwestern University. The experiments were performed on both the paretic and non-paretic side of a total number of 34 stroke survivors.

The following inclusion criteria were applied: stable medical condition, absence of aphasia or significant cognitive impairment, absence of motor or sensory deficits in the non-paretic side, absence of severe muscle wasting or major sensory deficits in the paretic limb, and spasticity in the involved ankle or elbow muscles for duration of at least 1 year.

### Clinical assessment

All stroke subjects were evaluated clinically using the MAS to assess muscle spasticity (range 1 to 5) [36] prior to each experiment by the same physical therapist, who had been well trained and had several years experience in MAS measurement.

The MAS was applied to the paretic joints of both the ankle and elbow.

Ashworth and Modified Ashworth scores are generated by manually manipulating the joint through its available range of motion and clinically recording the resistance to passive movements. In other words, the examiner seeks to assess how joint stiffness changes with joint position and velocity.

### Apparatus

**Figure 1:**
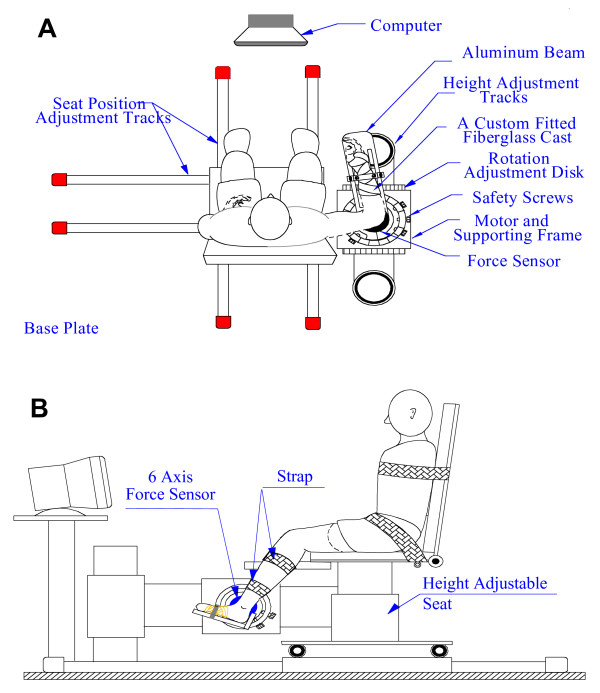
**Experimental Apparatus**. The upper (panel A) and lower (panel B) extremity apparatuses including the joint stretching motor device, the height adjustable chair, and force and position sensors.

For the elbow study, subjects were seated on an adjustable, chair with their forearm attached to the beam of a stiff, position controlled motor by a custom fitted fiberglass cast (Fig. 1A). For the ankle study, subjects were seated with the ankle strapped to the footrest and the thigh and trunk strapped to the chair. The seat was adjusted to provide shoulder abduction of 80°, or knee flexion of 60°, and align the joint axis of the rotation with axis of the torque sensor and the motor shaft (Fig. 1B).

### Recordings

The joint stretching motor device operated as a position control servo driving elbow or ankle position to follow a command input. Joint position was recorded with a precision potentiometer. Torque was recorded using a 6-degree of freedom load cell and velocity was recorded by a tachometer for both experiments.

In ankle joint studies, displacements in the plantar-flexion direction were taken as negative and those in the dorsi-flexion direction as positive, while in elbow joint, displacements in the flexion direction were taken as negative and those in the extension direction as positive. Also, a 90° angle of the elbow and ankle joint was considered to be the neutral position (NP) and defined as zero.

Electromyograms (EMGs) from tibialis anterior and lateral gastrocnemius for ankle joint and from biceps,  brachioradialis, and triceps for the elbow were recorded using bipolar surface electrodes (Delsys, Inc. Boston, MA). Position, torque, and EMGs were filtered at 230 Hz to prevent aliasing, and sampled at 1 kHz by a 16 bit A/D.

### Experimental procedures

Ankle and elbow passive Range of Motion (ROMs) were measured with the subjects attached to the motor, but with the motor turned off. Their ankle and elbow joints were manually taken through maximum range (plantar and dorsi-flexion, and flexion and extension, respectively). The typical angular range was from 50° plantar-flexion to 20° dorsi-flexion for ankle joint and from 45° flexion to 75° extension for the elbow joint.

To evaluate the stretch reflex response and to measure reflex torque magnitude, a series of 10 pulses was applied to the elbow/ankle joint with displacement amplitude of 5 deg and width of 40 ms. Pulses were applied with the joint placed in the neutral position and the responses ensembled-averaged.

To identify overall stiffness properties and to separate the reflex and intrinsic components, we used Pseudorandom Binary Sequence (PRBS) inputs with amplitude of 0.03 rad and a switching interval of 150 ms. Our previously published results demonstrated that these perturbations are appropriate to characterize the joint dynamic stiffness at each functional condition and to separate its intrinsic and reflex components [33]. Also, they are well tolerated by the people with spasticity [32-35,37].

Trials were conducted at different joint positions from full plantar-flexion to maximum tolerable dorsi-flexion, with 5 degree intervals for ankle joint and from full flexion to maximum extension, with 15 degree intervals for elbow joint. Each position was examined under passive conditions, where subjects were instructed to remain relaxed. Following each trial, the torque and EMG signals were examined for evidence of non-stationarities or co-activation of other muscles. If there was evidence of either, the data were discarded and the trial was repeated.

### Analysis procedures

**Figure 2:**
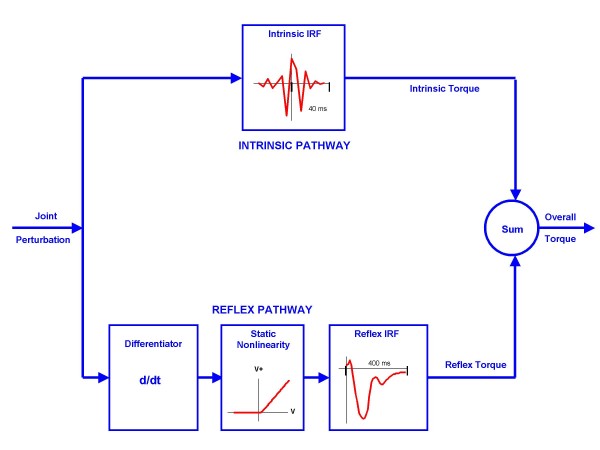
**Parallel Cascade System Identification Model**. The parallel cascade structure used to identify intrinsic and reflex stiffness. Intrinsic dynamic stiffness is represented in the upper pathway by the intrinsic stiffness impulse response function. Reflex dynamic stiffness is represented by the lower pathway as a differentiator, followed by a static nonlinear element and then a linear impulse response function. The nonlinear element is a half wave rectifier which shows the direction of stretch. Filled areas show reflex torque. V represents perturbation velocity. V+ represents half wave rectified velocity.

We used a parallel cascade system identification technique to identify reflex and intrinsic contributions to elbow/ankle dynamic stiffness. This technique, described in detail in earlier publications [33,38], is explained further in Figure 2.

Intrinsic stiffness (top pathway) was estimated in terms of a linear Impulse Response Function (IRF), which is a curve relating position and torque. The IRF characterizes the behavior of the system over its entire range of frequencies. The reflex pathway (bottom pathway) was modeled as a differentiator in series with a delay, a half-wave rectifier (indicating the direction of stretch), and a dynamic linear element. Reflex stiffness was estimated by determining the IRF between half-waved rectified velocity as the input and reflex torque as the output. The intrinsic and reflex stiffness IRFs were convolved with the experimental input to predict the intrinsic and reflex torque, respectively.

IRFs were assessed in terms of the percentage of the output (torque) variance accounted for (%VAF), defined as:

(1) $$\%VAF=100*\{1-\sum_{1}^{N}{\left(TQ-\hat{T}Q\right)}^{2}/\sum_{1}^{N}TQ^{2}\}$$

where, N: the number of points, *TQ*: the observed torque, $\hat{T}Q$: the torque predicted by the IRF

Intrinsic and reflex stiffness gains were calculated by fitting linear models to their IRF curves.

### Statistical analysis

Standard t-tests procedures were used to test for significant changes in intrinsic and reflex stiffness between paretic and non-paretic joints. Results with *p *values less than 0.05 were considered significant.

Spearman correlation coefficients were computed to test the relationship between the stroke effects on intrinsic and reflex stiffness gains and Ashworth scores in the spastic, paretic elbow and ankles.

## Results

### Joint reflex torque and Ashworth

**Figure 3:**
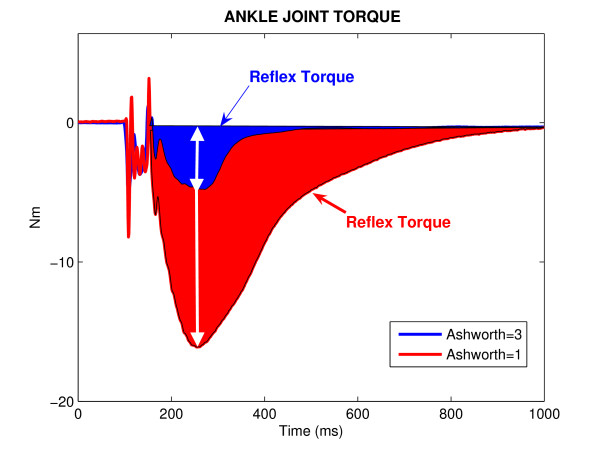
**Joint Torque**. Ankle joint torque for two different hemiparetic spastic subjects with different Ashworth-scores.

Figure 3 shows a typical position pulse trial with displacement amplitude of 5 deg and width of 40 ms, which stretched the ankle joint around the neutral position. The ankle torque induced by this stretch is shown for the paretic limbs of two people with stroke, each with different degree of spasticity (Fig. 3).

There are two distinct components to the torque response; a torque increase correlated with ankle position and its derivatives, beginning with no delay attributed to intrinsic mechanics, and a transient component associated with dorsiflexion displacements only, likely representing the contribution of stretch reflex mechanisms. The colored area reflects the integral of the torque response elicited by the rising edge of the pulse perturbation-it's a good (if indirect) estimate of reflex gain. Unexpectedly, both peak-reflex torque and reflex gain were larger in the subject with MAS score of 1 than in the subject with the MAS score of 3.

### Correlations between stroke effects on neuromuscular properties and Ashworth score

Beginning a short time after measuring the MAS, we quantified intrinsic stiffness (*K*) and reflex stiffness (*G*_*R*_)at the neutral positions (the joint angle of 90°) around which elbow flexor reflexes, and ankle plantarflexor reflexes are expected to have their maximum relative contributions to overall stiffness [35].

We then looked for correlations between our objective measures of dynamic joint stiffness and clinical assessment of muscle tone, the MAS.

The stroke effects on each neuromuscular property (i.e., *K *and *G*_*R*_) were then estimated as the difference in each property between the paretic and non-paretic joints.

**Figure 4:**
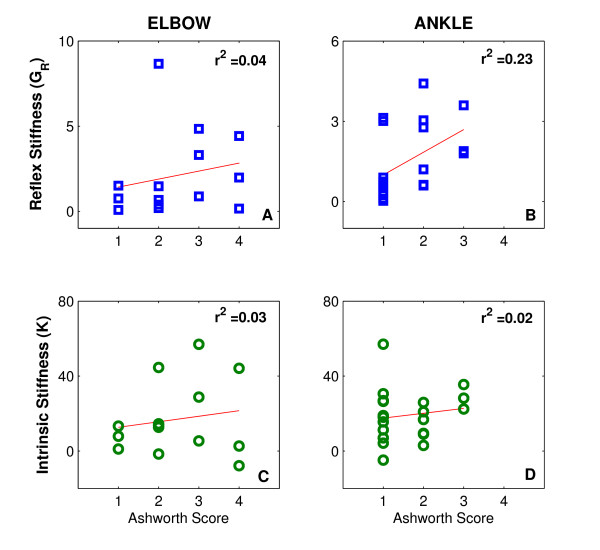
**Intrinsic and Reflex Stiffness vs Modified Ashworth Scale**. Scatter plots of stroke effects on reflex (G_R_) and intrinsic stiffness (K) for both elbow (left column) and ankle (right column) versus the values of the Modified Ashworth Scale (MAS).

Figure 4 shows scatter plots for stroke effects on *G*_*R *_(top row) and on *K *(left row) versus the values of MAS for the elbow (left column) and ankle (right column). The scatter of the points and the low values of the correlation coefficient (r^2 ^< 0.23) indicate that there was no significant relation between our objective quantitative measures of stroke effects on joint neuromuscular properties and the clinical assessment of muscle tone (via the MAS).

### Position dependency of neuromuscular abnormalities

To further explore the possible correlation between our neuromuscular measures and the MAS, we also investigated the overall position-dependency of stroke effects; i.e. the differences between paretic and non-paretic sides as the starting joint angle were changed systematically.

To ensure that the amplitudes of the reflex EMG and torque responses did not change with time, or as a result of the perturbation stimuli, pulse trials were injected before and after PRBS trials and the responses were compared. Torque and EMGs were recorded and ensemble-averaged. Changes in reflex torque of more than 20% before and after trials were taken as evidence of a change in the subject's state, due to fatigue or other factors, and trial was discarded. This occurred rarely and in most experiments no trials were discarded.

**Figure 5:**
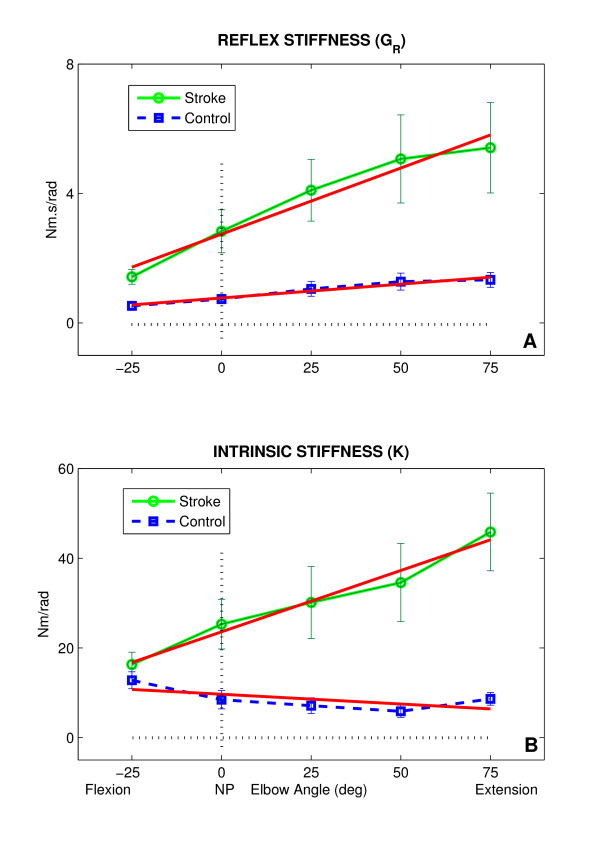
**Intrinsic and Reflex Stiffness vs Joint Angle**. Modulation of reflex and intrinsic stiffness as a function of elbow position for paretic and non-paretic groups. Group results ± SD.

Figure 5 shows group average results for modulation of *G*_*R *_and *K *as a function of elbow position over the ROM for both paretic and non-paretic limbs. *G*_*R *_was significantly larger in the paretic than non-paretic elbow at most positions (p < 0.0001) and the difference increased as the elbow was extended (Fig. 5A). Position dependence was similar in both groups; the reflex stiffness gain continuously increased from full flexion to full extension. However, the rate of change was larger in the paretic than in the non-paretic limb.

Similar to *G*_*R*_, *K *was significantly larger in the paretic than in the non-paretic limb at extended joint positions (p < 0.001). *K *was strongly position dependent although this dependency was different for both sides. *K *increased sharply in the paretic limb as the elbow was moved from mid-flexion to full extension, whereas it decreased slowly and remained invariant in the contralateral limb.

The position-dependency of both *G*_*R *_and *K *is well described by a first-order model as indicated by the superimposed solid lines (r^2 ^> 0.81, p < 0.001). The stroke effects on reflex and intrinsic mechanical properties were estimated using the difference between the slope/intercepts of the paretic and the slope/intercepts of the non-paretic side. Since abnormalities in the *G*_*R *_and *K *of the ankle joint in people with stroke were basically similar to those of the elbow joint [35], the stroke effects on these mechanisms were estimated similarly, but the related plots are not shown for the ankle.

### Correlations between position dependency of mechanical abnormalities and Ashworth score

We examined the position-dependent data for each of reflex and intrinsic mechanisms at each elbow and ankle joint separately. To correlate our results with this scale, we estimated the stroke effect on these joint mechanical properties (as shown in Figure 5) and calculated the linear relationships between the calculated slopes and intercepts with the Modified Ashworth scores.

**Figure 6:**
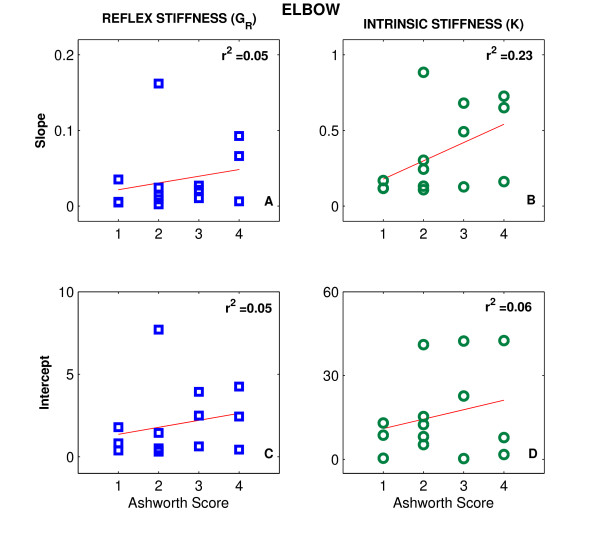
**Intrinsic and Reflex Slopes and Intercepts vs Modified Ashworth Scale for Elbow**. Scatter plots of the slope (top row) and intercepts (bottom row) for G_R _and *K *versus the values of the Modified Ashworth Scale (MAS) for the elbow.

**Figure 7:**
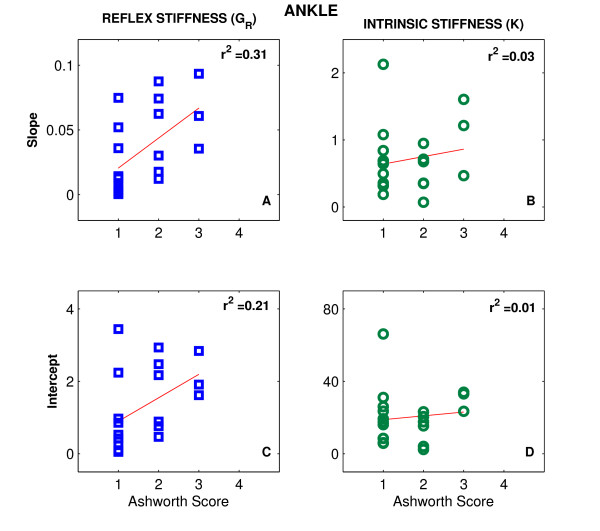
**Intrinsic and Reflex Slopes and Intercepts vs Modified Ashworth Scale for Ankle**. Scatter plots of the slope (top row) and intercepts (bottom row) for G_R _and *K *versus the values of the Modified Ashworth Scale (MAS) for the ankle.

Figures 6 and 7 show scatter plots of the slope and intercepts for G_R _(left column) and *K *(right column) versus the values of MAS for the elbow (Fig. 6) and ankle (Fig. 7), respectively. The scatter of the points and the low values of the correlation coefficient (r^2 ^< 0.31, p < 0.01) indicate that there was no significant relation between these variables, and consequently between our quantitative measures of stroke effects on joint mechanics and the MAS.

### Validity of the parallel-cascade model

**Figure 8:**
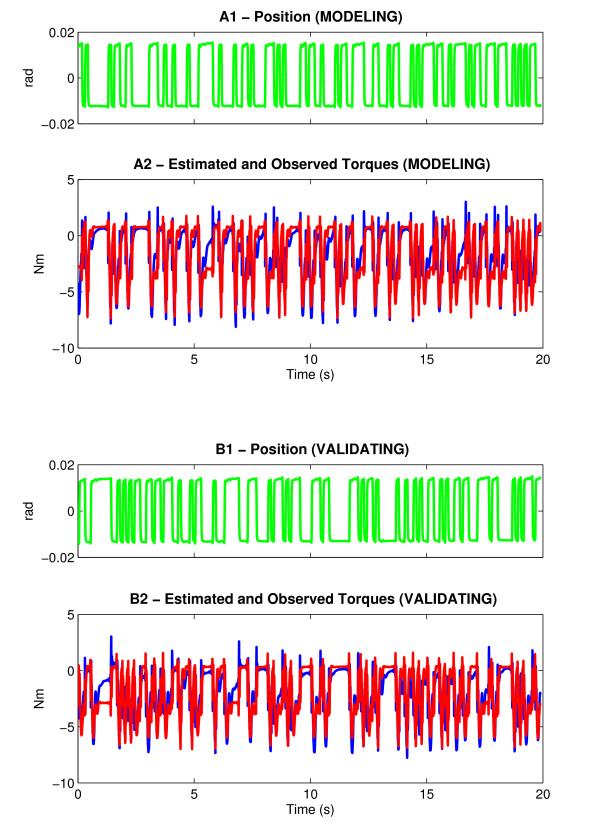
**Validation of the Parallel Cascade System Identification Model**. Data used for system identification modeling (panel A), data used for testing the validity of the model (panel B). In panel A2, the actual torque response (in blue) to PRBS input (Panel A1), and the predicted torque (in red), are superimposed. This prediction was derived by convolving the impulse response with the position input. A different PRBS input (panel B1) was used, and convolved with the first IRF. The predicted torque (Panel B2 – red) matches closely to the actual torque (Panel B2 – blue) recorded in this series.

In our earlier studies, we demonstrated that the parallel-cascade model is valid and reliable for both upper and lower extremity and for normal and spastic subjects including SCI and stroke subjects [32-35,37]. However, to further validate our technique in this paper, we applied two PRBS sequences in succession with the same initial conditions to a stroke subject. Using the parallel cascade model, we estimated intrinsic and reflex IRFs and predicted the overall torque. Fig. 8-A2 shows the predicted torque (red lines) superimposed on the recorded torque (blue lines). The %VAF of fit was 92.6% indicating a very good match.

To further assess validity we convolved the intrinsic and reflex stiffness IRFs (obtained from trial 1) with the PRBS input of trial 2 (Fig. 8-B1) to estimate the overall torque. Again, the overall predicted torque (Fig. 8-B2, red lines) describes accurately the actual recorded torque (blue lines). The %VAF of fit was 88.9%, which was about 4% smaller than that of trial 1 demonstrating the validity of this model for this data set. Similar results were obtained in a randomly selected group of 5 subjects.

## Discussion

Our earlier studies demonstrated that both neural and muscular systems are altered in spastic limbs, but the changes were complex and depended on multiple factors. In the current study, we compared the changes in intrinsic and reflex stiffness at different joint angles in both upper and lower extremities with a standard clinical manual assessment of spasticity (Modified Ashworth). Our main result was that there was no significant relation between our quantitative measures of stroke effects on spastic joints and the clinical assessment of muscle tone, as reflected in the Ashworth scores.

Although the Ashworth scales have often been used clinically, the question of its utility as a prognostic measure has not been fully addressed. This issue is important when these assessments are used to choose the appropriate treatment or as outcome measures following intervention, both clinically and for research.

### Neuromuscular abnormalities

Our findings in people with chronic hemiparetic stroke demonstrate that both reflex and intrinsic stiffnesses in elbow and ankle joints are strongly dependent on position, similar to our previous findings in SCI subjects [34]. Furthermore, reflex stiffness (*G*_*R*_) was significantly larger in paretic than in the non-paretic side at most positions. However the position-dependence of reflex stiffness was broadly similar in both groups. Although the rate of change was a distinguishable feature between paretic and non-paretic sides, it was significantly greater in the paretic than in the non-paretic limb.

Given these observations, the question arises as to how an ordinal scale like MAS can distinguish the rate of changes at different joint positions. While Ashworth scales give us one ordinal score to define joint spasticity, they certainly can't represent the joint dynamic stiffness and position- and velocity dependency of both intrinsic and reflex components.

### Neuromuscular abnormalities and modified Ashworth scale (MAS)

In our present study, we have investigated the biomechanical parameters of stretch reflex responses and their correlation with available spasticity scales. The Ashworth Scale produces a global assessment of the resistance to passive movement of an extremity, not just stretch-reflex hyperexcitability. Specifically, the Ashworth score is likely to be influenced by non-contractile soft-tissue properties, by persistent muscle activity (dystonia), by intrinsic joint stiffness, and by stretch reflex responses [39]. Our results reveal that there is no significant correlation between reflex torque at joint and MAS scores by measuring peak torque and area under the reflex torque curve.

Others have reported different results in broadly similar studies. Starsky et al. (2005) showed that biomechanical parameters, especially peak reflex torque at the highest speed, had a strong correlation with the AS. They suggested that the Ashworth measurements of spastic hypertonia are influenced strongly by stretch reflex hyperexcitability [40]. The differences between our results and Starsky et al. group can potentially be explained by different techniques that we have applied. They used several assumptions and simplifications that may result in over- or under- estimation of reflex torque. For example, Starsky et al. (2005) assumed that slow angular velocities effectively eliminate viscous contributions to joint torque. We believe this assumption to be inaccurate, because muscle and passive tissues will each show viscous behavior, independent of added reflex action. In addition, these authors assumed linearity for the angular relation between reflex torque and joint angle, whereas we (and others) have shown that these relations are highly non-linear [33,38]. Thus, reflex stiffness, which is a dynamic relation between reflex torque and velocity [33,38], cannot be simply estimated by taking the derivative of reflex torque with respect to joint angle, as was done by these authors.

Given the known velocity dependence of the spastic reflex response, the control of the stretching velocity for the MAS in clinical practice is another controversial problem. Indeed, different investigators recommend different velocities to determine MAS or AS score but there is not consistency in these recommendations to date. To overcome this problem, recently, it has been suggested that other clinical scales like the Tardieu Scale which involves the use of two speeds of passive movement (one very slow, the other as fast as possible) could be superior than MAS in identifying the neural component of spasticity [41].

A groundwork study of upper-limb reflex responses performed by Wolf et al. (1996) suggested that the reflex response onset threshold might also depend on speed. They demonstrated that the most consistent reflex responses were obtained at the faster speeds (1.0 radian/s) when starting at the more flexed position (90 degree) [42]. The speed-dependence of the reflex response for all reflex-EMG parameters and the torque variable is consistent with previous studies utilizing ramp-and-hold extensions at the elbow [43,44]. It follows that angular velocity is an important parameter, which requires rather precise control.

While the position dependence of stretch reflex is one of the defining characteristics of spastic hypertonia, the question about which angular range should be used to distinguish the reflex effects from intrinsic effects has not been identified. With our approach, we were unable to detect a correlation between spasticity measures and MAS scores. Taken together we strongly believe that the MAS score doesn't give us any information about spasticity producing factors or contributing components.

## Conclusion

Our findings revealed that there was no significant correlation between the quantitative measures of neural and muscular components of joint dynamic stiffness and MAS scores, for either the upper extremity or the lower extremity. These findings indicate that Modified Ashworth scores are quite inconsistent with more objective measures of spasticity. Consequently, although the MAS seems to be a quick and easy clinical test to assess spasticity, and it remains widely accepted, it neither can characterize the contributions of neuromuscular components to spasticity nor their modulation with position and velocity of the joint stretch.

If the purpose of a clinical assessment is to measure response to an intervention, it is important to distinguish the mechanical and neurogenic components of spasticity. Accordingly, the MAS might be used as a clinical measurement of muscle tone alongside other precise measures but it is not sufficient alone for monitoring the development of spasticity or progress of treatment.

## Abbreviations

ROM: Range of Motion; MAS: Modified Ashworth Scale; EMGs: Electormygrams; PRBS: Pseudorandom Binary Sequence; IRF: Impulse Response Function; VAF: Variance Accounted for; G_*R*_: Reflex stiffness gain; K: Intrinsic stiffness gain; NP: Neutral Position.

## Competing interests

The authors declare that they have no competing interests.

## Authors' contributions

LA participated in performing the experiments, interpreting data and writing the paper. WZR participated in interpreting data and writing the manuscript, RLH participated in interpreting data and writing the manuscript, and MMM designed the study, supervised data collection and analysis, and participated in interpreting and writing the manuscript. All authors read and approved the final manuscript.
